# Superselective Lipiodol CT-guided Microwave Ablation for Small Hepatocellular Carcinoma: A Novel Imaging Guidance Strategy

**DOI:** 10.2174/0115734056467384260327043315

**Published:** 2026-04-02

**Authors:** Yun-Liang Wang, Ya-Pan Guo, Lei Li, Xin-Guo Zhu, Tong-Qing Xue, Zhi Li

**Affiliations:** 1 Department of General Surgery, The First Affiliated Hospital of Soochow University, No. 188 Shizi Street, Suzhou, Jiangsu 215006, China; 2 Department of Interventional Radiology, Suzhou Xiangcheng People's Hospital, No. 1060 Huayuan Road, Suzhou 215131, China; 3 Department of Hepatological Surgery, Suzhou Xiangcheng People's Hospital, No. 1060 Huayuan Road, Suzhou 215131, China; 4 Department of Pain and Intervention Management, Huai'an Clinical Medical College of Jiangsu University & Huai'an Hospital of Huai'an City, Huai'an, Jiangsu 223000, China; 5 Department of Interventional Radiology, The First Affiliated Hospital of Soochow University, No. 188 Shizi Street, Suzhou, Jiangsu 215006, China

**Keywords:** Hepatocellular carcinoma, Microwave ablation, Computed tomography, Lipiodol, Image-guided ablation, Non-contrast CT (NCCT), Local tumor progression (LTP)

## Abstract

**Introduction:**

Microwave Ablation (MWA) is a well-established curative therapy for early-stage Hepatocellular Carcinoma (HCC). However, conventional Non-Contrast CT (NCCT) guidance for MWA faces limitations, including poor visualization of isodense lesions and significant needle artifacts, which can compromise targeting accuracy. This study evaluated a novel imaging-guidance strategy utilizing superselective lipiodol CT to explore its feasibility in addressing these challenges.

**Materials and Methods:**

Treatment-naive patients with BCLC 0/A HCC within the Milan criteria were included in this retrospective study. All patients underwent superselective transarterial lipiodol-only marking followed by CT-guided MWA within 1 week. Treatment efficacy was evaluated based on technical success, Local Tumor Progression (LTP), Intrahepatic Distant Recurrence (IDR), Recurrence-Free Survival (RFS), and Overall Survival (OS). Imaging-guidance performance was descriptively evaluated based on intraprocedural lesion visualization and operator-adjusted window settings used to facilitate needle positioning.

**Results:**

Forty-five patients with 57 lesions were enrolled. The mean lesion diameter was 18.2 ± 8.8 mm. The initial technical success rate was 95.5% (43/45). The 1-, 2-, and 3-year rates were: LTP: 2.2%, 4.4%, 4.4%; IDR: 24.4%, 37.7%, 44.4%; RFS: 68.9%, 52.7%, 38.2%; OS: 85.1%, 72.4%, and 60.5%. During lipiodol CT-guided procedures, lesions that were isodense and poorly visualized on pre-procedural NCCT were identifiable to the operator, and the window level settings used for final needle positioning confirmation had a mean value of 252.2 ± 75.8 Hounsfield Units.

**Discussion:**

The superselective lipiodol CT guidance strategy demonstrated feasibility in addressing several practical limitations of conventional NCCT. It allowed visualization of otherwise inconspicuous isodense lesions, supported needle positioning under reduced metallic artifact conditions at empirically adjusted window levels, and aided the visualization of adjacent portal vein branches during the procedure. This approach utilizes Lipiodol primarily as a long-lasting contrast agent for enhanced CT visualization, rather than for its chemotherapeutic or embolic effect.

**Conclusion:**

Superselective lipiodol CT guidance is a feasible imaging approach for intraprocedural lesion visualization and needle positioning during MWA of small HCC in selected procedural scenarios. Controlled comparative or quantitative validation studies are warranted.

## 1 INTRODUCTION

Microwave Ablation (MWA) is a well-established curative therapy for early-stage Hepatocellular Carcinoma (HCC) [1, 2]. For early-stage HCC, MWA is a guideline-endorsed curative-intent option, with outcomes approaching those of surgical resection in appropriately selected patients [3, 4]. Percutaneous MWA is commonly guided by Ultrasound (US), Computed Tomography (CT),orMagneticResonanceImaging(MRI)[5-7]. While the US allows for real-time needle tracking, CT provides more intuitive and comprehensive anatomical images and is less susceptible to acoustic interference, making it the preferred guidance modality in many interventional radiology departments [8].

However, conventional Non-Contrast CT (NCCT) guidance has several limitations for targeting liver tumors. Firstly, isodense lesions are often poorly visualized or entirely invisible on NCCT. Secondly, MWA needles produce significant metal artifacts on CT, which can hinder the accurate assessment of the needle tip position relative to the lesion boundaries. Thirdly, portal or hepatic vein branches are inadequately delineated on NCCT, increasing the risk associated with ablating lesions situated near the hepatic hilum.

In an attempt to address these limitations inherent to NCCT, we employed a superselective lipiodol CT guidance strategy, which involves superselective transarterial lipiodol-only marking, followed by CT-guided MWA within 1 week. While the combination of Transarterial Chemoembolization (TACE) and ablation has been extensively reported [9-11], our approach distinctly utilizes lipiodol primarily as a long-lasting contrast agent for enhanced CT visualization, rather than for its chemotherapeutic or embolic effect [12]. This article reports the technical and imaging guidance characteristics of this approach, with particular emphasis on its observed performance in visualizing isodense lesions, managing needle-related artifacts, and delineating adjacent critical structures.

## 2 MATERIALS AND METHODS

### 2.1 Study Design and Patients

This single-center, retrospective study was approved by the Institutional Review Board of The First Affiliated Hospital of Soochow University (Code: 2024-641). Due to its retrospective nature, the requirement for written informed consent from participants was waived by the board. Consecutive patients who underwent MWA for HCC at our center between January 2019 and December 2023 were screened for inclusion.

Given the retrospective, exploratory nature of this feasibility study, no formal sample size calculation was performed, and the sample size was determined by the number of consecutive eligible patients treated during the study period.

Inclusion Criteria: (1) Confirmed diagnosis of HCC, meeting clinical diagnostic criteria or confirmed by biopsy. (2) No prior locoregional or systemic therapies for recurrent HCC lesions, including ablation, TACE, hepatic arterial infusion chemotherapy, targeted therapy, or immunotherapy. Prior surgical resection with subsequent intrahepatic recurrence was permitted. (3) BCLC stage 0 or A within the Milan criteria. (4) Underwent the defined protocol of superselective lipiodol-only marking followed by MWA. (5) Availability of complete follow-up data, meaning the patient has available post-procedure follow-up imaging and clinical data for analysis.

Exclusion Criteria: (1) Simultaneous superselective lipiodol-only marking and cone-beam CT (CBCT)-guided MWA. (2) Patients who did not undergo MWA within 1 week of the superselective lipiodol-only marking.

### 2.2 Patient Screening and Enrollment

A total of 180 consecutive patients who underwent MWA for HCC were initially screened. The detailed screening process, including the number of patients excluded at each step and the specific reasons for exclusion, is presented in Fig. (**1**).

Patients were sequentially excluded for not meeting basic clinical criteria, for having received prior treatments, or for not undergoing the predefined superselective lipiodol-only marking workflow followed by CT-guided MWA within the specified time window. Ultimately, 45 patients with 57 lesions met all inclusion criteria and constituted the final study cohort.

### 2.3 Lesions Characteristics

Lesion location was recorded according to the Couinaud liver segment classification. High-risk locations were defined as lesions situated < 5 mm from critical structures, including the diaphragm, heart, large vessels (> 3 mm diameter), bile ducts, and gastrointestinal tract. This determination was made by consensus review of the pre-procedural cross-sectional imaging (CT/MRI) by two radiologists (with 8 and 12 years of experience). The measurement was performed using electronic calipers on the institutional picture archiving and communication system.

On baseline NCCT, isodense and poorly visualized lesions were defined as lesions with attenuation similar to or slightly lower than the surrounding liver parenchyma, resulting in insufficient lesion-to-liver contrast. Assessment of lesion conspicuity was performed by the operating interventional radiologist during procedural planning.

### 2.4 Superselective Lipiodol-only Injection

Procedures were performed *via* femoral or radial artery access under local anesthesia. An initial two-dimensional Digital Subtraction Angiography (DSA) of the proper hepatic artery was performed. Cone-Beam CT (CBCT) (Siemens Healthineers, Germany) with embolization navigation software (Syngo Embolization Guidance, Siemens Healthineers) was used to identify the tumor-feeding arteries. A 2.7F microcatheter (Progreat, TERUMO, Japan) was superselectively advanced into the subsegmental or finer tumor-feeding artery, avoiding branches supplying normal parenchyma. Repeat DSA was performed. If tumor staining was visible, pure Lipiodol (Lipiodol Ultra-Fluide, Guerbet, France) was slowly injected under fluoroscopic guidance. For hypovascular lesions without evident staining, a repeat CBCT was performed to confirm the microcatheter position and that the lipiodol distribution matched the pre-procedural CT/MRI lesion location.

The injection endpoint was defined as focal, dense lipiodol accumulation within the tumor bed, confirmed on real-time fluoroscopy and immediate post-injection CBCT, with minimal or no spillover into the surrounding non-tumorous parenchyma. The primary goal of the procedure was to mark the tumor rather than achieve complete embolization. While pure lipiodol can have a transient embolic effect, no deliberate stasis of blood flow was sought, and no solid embolic agents or chemotherapeutic drugs were used. The volume of Lipiodol was not predetermined but was titrated intra-procedurally under fluoroscopic and CBCT guidance. The injection was performed by a senior interventional radiologist with over 10 years of experience in TACE.

### 2.5 Microwave Ablation (MWA) Procedure

Microwave ablation was performed within 1 week after lipiodol-only marking under local anesthesia, guided by non-contrast CT using a 16-slice multidetector CT scanner (Philips Brilliance 16, Philips Healthcare, the Netherlands). The CT acquisition parameters were as follows: tube voltage 120 kV, automatic tube current modulation with a reference mAs of 150, rotation time 0.75 s, pitch 0.938, and collimation of 16 × 0.75 mm. Images were reconstructed using a standard Body Kernel (B) with a slice thickness of 5 mm for planning and 2 mm for intraprocedural guidance. Radiation exposure was managed in accordance with institutional CT protocols for interventional procedures, with dose parameters monitored by the scanner system. The CT acquisition and reconstruction protocol was applied consistently across all patients throughout the study period.

Pre-procedural intramuscular pethidine was administered for analgesia. Patients were positioned supine or lateral on the CT scanner table. Self-made longitudinal skin markers (cotton swabs) were placed over the liver area. A pre-procedural CT scan was obtained to determine the optimal puncture site and trajectory. Lesion detectability on lipiodol CT was assessed intraoperatively by the operating interventional radiologist, based on whether lesions previously considered isodense or poorly visualized on baseline NCCT could be visually identified and targeted during the procedure.

After local disinfection and anesthesia, the anesthetic needle (a 5 ml syringe) was left in the subcutaneous tissue as a marker. A reduced-Field-of-View (FOV) CT scan was then performed to confirm the correct skin entry point and trajectory. Using a stepwise approach, the MWA antenna (MaxBlate KY-2000A, Canyon Medical Inc., China) was advanced incrementally. This involved advancing the needle 2-5 cm, followed by a confirmatory CT scan (2 or 5 mm slice thickness) to verify the trajectory. This “advance-and-check” process was repeated 2-3 times until the needle tip reached the target. During needle advancement, the Window Level (WL) and Window Width (WW) were adjusted intraoperatively by the operating interventional radiologist to facilitate procedural visualization, such that the MWA needle shaft and tip could be visually discerned while the Lipiodol-marked target lesion remained identifiable, without the use of a predefined protocol or quantitative optimization algorithm. The WL and WW settings used for the final needle positioning confirmation were recorded. The mean WL was calculated from these recorded values.

Ablation commenced once the antenna tip was positioned 3-5 mm beyond the distal lesion margin. For subcapsular lesions, the overlying liver capsule was anesthetized using a 21-G needle infiltrated with lidocaine. MWA was performed using a MaxBlate KY-2000A antenna (Canyon Medical Inc., China). Ablation power ranged from 50 to 60 W, with duration typically set to 4-5 minutes per placement, adjusted based on lesion size. The number of placements varied depending on the lesion, with multiple placements used to ensure complete ablation. A 1-1.5 cm safety margin beyond the lesion was targeted to ensure effective necrosis. Upon completion, the antenna was withdrawn while applying 30W power to cauterize the needle tract. Post-procedure, non-contrast CT was performed to check for immediate complications.

### 2.6 Follow-up and Outcome Measures

Follow-up imaging was performed at 1 month, 4 months, and 7 months post-treatment, and then every 6 months thereafter (with a permissible window of ±10 days). The imaging modality (contrast-enhanced CT, MRI, or Contrast-Enhanced Ultrasound [CEUS]) was selected based on clinical routine, patient-specific factors (*e.g.*, renal function, contraindications), and scheduling logistics. To ensure consistent outcome assessment, all follow-up images were reviewed and adjudicated in consensus by two experienced abdominal radiologists. The final follow-up date for data analysis was October 31, 2025. Outcome measures were defined as follows:

Lesion-based analysis was used to assess the primary outcome of technical success, defined as the absence of any enhancing tissue at the ablation site on 1-month post-MWA contrast-enhanced imaging. If the lesion was not fully ablated, repeat ablation was performed. Local Tumor Progression (LTP) was defined as tumor recurrence directly adjacent to the ablation zone, and Intrahepatic Distant Recurrence (IDR) as tumor recurrence > 2 cm away from the ablation zone or within non-tumorous liver parenchyma.

Patient-based analysis was used for survival outcomes, including Recurrence-Free Survival (RFS), defined as the time from treatment to disease recurrence, death, or last follow-up, and Overall Survival (OS), defined as the time from first MWA to death or last follow-up. Complications were graded according to the Society of Interventional Radiology (SIR) guidelines.

### 2.7 Statistical Analysis

Statistical analysis was performed using SPSS software (version 26.0, IBM, USA). Continuous variables are expressed as mean ± standard deviation; categorical variables are presented as frequencies and percentages. Cumulative incidence rates for LTP, IDR, RFS, and OS at 1, 2, and 3 years are presented descriptively.

## 3 RESULTS

### 3.1 Patient and Lesion Characteristics

Forty-five patients (35 male, 10 female; mean age 59.9 ± 10.2 years) with 57 lesions were included. The Hepatitis B surface antigen (HBsAg) positivity rate was 86.7% (39/45). The mean lesion diameter was 18.2 ± 8.8mm. Lesions in high-risk locations constituted 22.8% (13/57). The volume of Lipiodol used ranged from 2 to 8 ml. The volume was titrated to achieve saturation of the tumor and a small surrounding safety margin, as visualized under fluoroscopy and CBCT. (Table **1**).

### 3.2 Imaging Guidance Efficacy

Among the 57 lesions, 26 (45.6%) were isodense on pre-procedural NCCT and were considered poorly visualized. On lipiodol CT, all lesions that were previously considered poorly visualized on baseline NCCT were identifiable to the operating interventional radiologist during the procedure (Fig. **2**). This assessment was operator-based and non-blinded and should be interpreted as a feasibility observation rather than diagnostic accuracy.

On lipiodol CT, MWA needle artifacts appeared reduced at window level settings ranging from 98 to 357 HU (mean 252.2 ± 75.8 HU), while the target lesion remained visible for procedural guidance (Fig. **3**). In seven cases, lesions were adjacent to secondary branches of the portal vein. On Lipiodol CT, lipiodol-enhanced liver parenchyma provided increased contrast relative to the portal vein branches, which facilitated their visualization and supported trajectory planning during the procedure (Fig. **4**).

### 3.3 Treatment Efficacy and Survival Outcomes

The initial technical success rate was 95.5% (43/45). The remaining 2 patients achieved complete ablation after a second MWA session, resulting in a final technical success rate of 100%. With a median follow-up of 28 months, the 1-, 2-, and 3-year cumulative rates were as follows: LTP: 2.2%, 4.4%, 4.4%; IDR: 24.4%, 37.7%, 44.4%; RFS: 68.9%, 52.7%, 38.2%; OS: 85.1%, 72.4%, and 60.5%. No SIR grade 3 or higher complications related to lipiodol-only marking or MWA occurred.

### 3.4 Safety and Complications

No SIR grade 3 or higher complications occurred. Minor adverse events included transient post-embolization symptoms such as low-grade fever, mild fatigue, and self-limited abdominal discomfort, as well as periprocedural pain requiring short-term oral or intravenous analgesia. These symptoms were managed conservatively and resolved without prolonged hospitalization.

Mild, transient elevations in liver function parameters were occasionally observed on routine post-procedural laboratory testing, without evidence of hepatic decompensation or biliary injury. No cases of liver abscess, bile duct injury, major vascular complications, or needle tract seeding were identified.

No angiography-related complications (including access-site hematoma, arterial dissection, or contrast-related adverse events) were documented, and no procedure-related readmissions occurred during the periprocedural period.

## 4 DISCUSSION

The principal finding of this study is that superselective lipiodol CT guidance is feasible for addressing certain practical limitations encountered during percutaneous MWA under conventional NCCT, particularly in lesion targeting and needle visualization.

Firstly, we observed that all lesions considered isodense/poorly visualized on baseline NCCT were identifiable to the operator on lipiodol CT during the procedure. This is consistent with the historical use of lipiodol CT for diagnostic lesion detection [13, 14], but here we repurpose lipiodol deposition primarily as an intraprocedural visualization aid. By using CBCT to achieve superselective catheterization [15-17], we maximize Lipiodol deposition in the target lesion while minimizing non-target parenchymal staining, resulting in a high-contrast target for subsequent CT guidance.

Secondly, we observed a range of window level settings (mean 252.2 HU) that were empirically used to reduce the visual impact of metallic artifacts while preserving visibility of the lipiodol-labeled lesion during the procedure. This dual visualization is critical for precise antenna placement, a challenge frequently encountered in conventional CT-guided ablation. It is important to note that lipiodol marking does not eliminate the metallic artifact from the MWA antenna. Instead, by creating a high-contrast, persistent target, it allows the operator to adjust the CT window level aggressively to suppress the visual interference of the artifact while the target lesion remains clearly visible. This facilitates a more confident assessment of the needle tip relative to the lesion. The reported window level range reflects an empiric, operator-dependent observation rather than a validated or reproducible imaging protocol, and interobserver agreement was not formally assessed in this study.

Thirdly, the parenchymal enhancement from Lipiodol provided improved contrast for visualizing second-order portal venous branches. While large vessels are visible on plain CT, the enhanced contrast is beneficial for recognizing smaller branches adjacent to the ablation zone, potentially enhancing procedural safety near the hilum.

Our approach differs fundamentally from most reported TACE-ablation combinations [18-20]. We deliberately used Lipiodol without chemotherapy or permanent embolic particles. The primary goal was not to achieve a synergistic tumoricidal effect, but to leverage lipiodol as a persistent imaging tracer. This concept is analogous to the use of fiducial markers or fluorescent dyes in other oncologic procedures, positioning our work as a refinement in image-guidance rather than in combination therapy.

Wu *et al*. previously demonstrated the efficacy of lipiodol CT-guided radiofrequency ablation [11]. Our study builds upon this concept by specifically detailing and validating the technical advantages of the superselective approach for MWA. We provide quantitative data on artifact reduction and qualitative evidence of improved structure delineation, which are paramount for interventional radiologists.

An ideal ablation guidance modality should fulfill the “4 See” criteria: See the lesion, See the needle, See the complications, and See the effect. While no single modality perfectly meets all criteria, our superselective Lipiodol CT technique may serve as a practical refinement in selected scenarios, particularly with respect to lesion visualization and needle visualization. It is important to note that this is a retrospective, single-arm feasibility study. The reported outcomes are descriptive and cannot establish causality or superiority over standard guidance methods.

The LTP, IDR, RFS, and OS outcomes reported in this study are descriptive for this selected cohort undergoing the specified protocol. Given the single-arm, retrospective design and the absence of a comparator group, these outcomes cannot be attributed to the Lipiodol-only marking or imaging guidance technique. Recurrence patterns and survival in HCC are strongly influenced by baseline liver disease, tumor biology, and subsequent treatments during follow-up. Controlled comparative studies are required to assess any causal relationship between the marking technique and oncologic outcomes.

While this technique may provide additional support for lesion visualization and needle guidance, it is important to recognize that the superselective Lipiodol marking step introduces additional angiographic procedures, with associated increases in radiation exposure, contrast use, procedural time, and cost. Accordingly, this approach is not intended as a routine alternative to conventional ultrasound- or NCCT-guided ablation. Rather, its potential value lies in selected problem-solving scenarios, such as lesions that are isodense on NCCT, poorly visualized on ultrasound, or located in anatomically challenging regions where precise needle placement is critical. In this context, the trade-off between improved visualization and added procedural burden should be carefully considered on a case-by-case basis.

This study has several limitations. Its retrospective, single-arm design with a modest sample size and the lack of a control group preclude comparative efficacy claims and limit the generalizability of the findings. The cohort represents a selected population defined by the superselective lipiodol-only marking workflow, and the predominance of hepatitis B etiology may further limit the generalizability of the findings, which should therefore be interpreted in this context. Methodologically, lesion visibility was assessed without a formal blinded review, and long-term outcomes (*e.g.*, RFS and OS) are reported as descriptive cumulative rates; therefore, no causal inference regarding oncologic outcomes can be drawn from this study. The key technical advantage of artifact reduction *via* window-level adjustment, while practical, was operator-dependent and not quantitatively validated. Finally, the additional angiographic marking procedure adds time, cost, and procedural risk, a trade-off that may not be justified for lesions already well-visualized by conventional means. Future prospective, randomized studies are warranted to validate the clinical value of this guidance strategy.

## CONCLUSION

Superselective Lipiodol CT guidance is a feasible imaging strategy for intraprocedural guidance during MWA of small HCC in selected procedural scenarios. This study provides a descriptive technical assessment of its intraprocedural use for lesion visualization and needle positioning and should be interpreted as a feasibility-oriented, hypothesis-generating report rather than evidence of comparative efficacy or oncologic outcome benefit. Further studies incorporating appropriate comparators or quantitative validation are required to define its clinical role.

## Figures and Tables

**Fig. (1):**
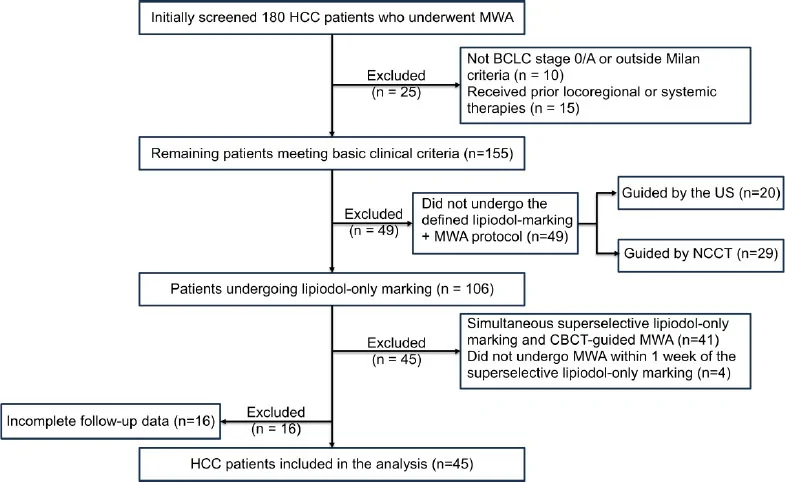
Patient enrollment flowchart.

**Fig. (2):**
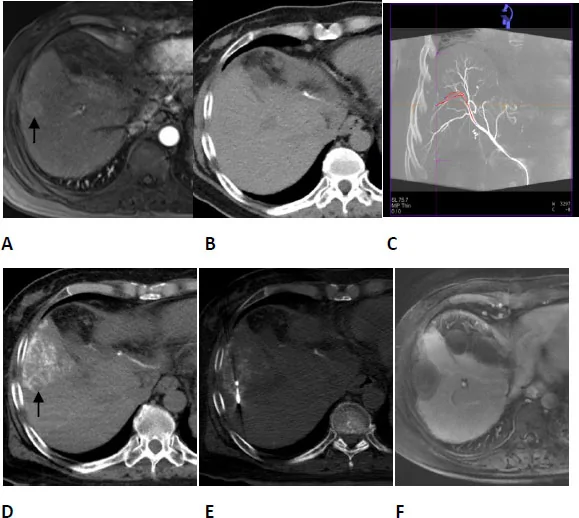
Case-based illustration of the procedural workflow for superselective lipiodol CT–guided microwave ablation of an isodense HCC lesion. The procedural sequence is illustrated from **A to F**. **A:** Pre-procedural MRI (arterial phase) demonstrates a lesion (arrow) in segment VI. **B:** The lesion is inconspicuous on pre-procedural non-contrast CT. **C:** Cone-beam CT (CBCT) acquired during superselective lipiodol-only marking. **D:** Subsequent lipiodol CT clearly delineates the hyperdense lesion (arrow), enabling CT-based targeting. **E:** CT-guided MWA antenna placement with reduced visual impact of needle-related artifacts at optimized window settings, while the lesion remains clearly visible. **F:** Contrast-enhanced MRI at 1 month post-ablation confirms complete necrosis.

**Fig. (3):**
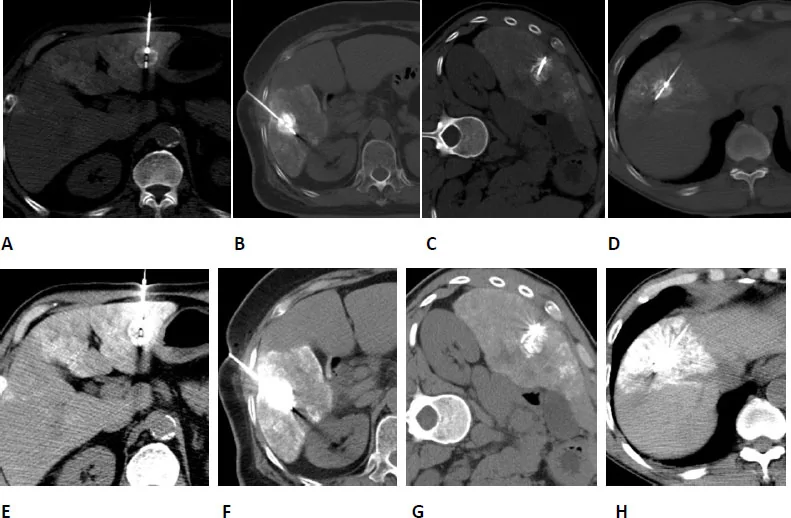
Side-by-side comparison of window level settings for MWA needle artifact reduction on superselective lipiodol CT. **A–D** (upper row) show four representative cases acquired with optimized window level settings, in which both the MWA antenna and the lipiodol-labeled lesions are clearly visualized. **E–H** (lower row) show the corresponding images of the same cases displayed using a standard abdominal window, where metallic needle artifacts obscure lesion visualization.

**Fig. (4):**
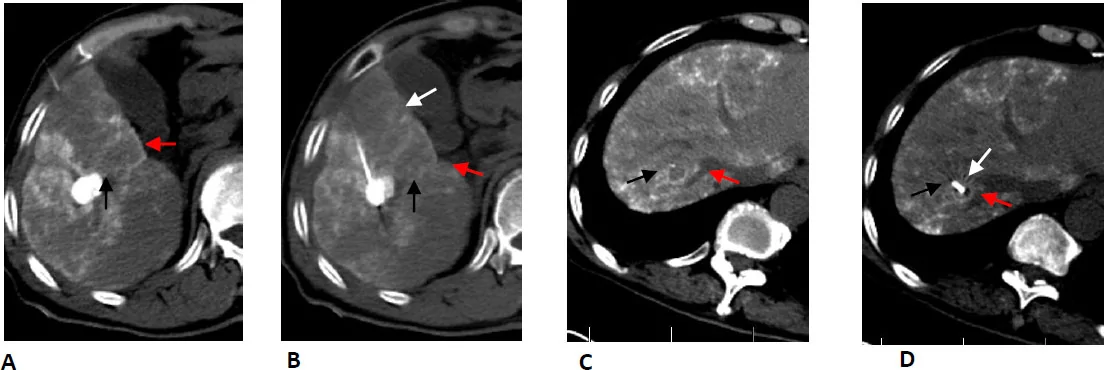
Representative examples illustrating enhanced visualization of adjacent vascular structures during CT-guided MWA using superselective lipiodol CT in high-risk locations. **A–B:** show a lesion located adjacent to portal vein branches, while C–D demonstrate a lesion adjacent to hepatic vein branches. **A, C:** Lipiodol CT clearly delineates target lesions (black arrows) and the adjacent hypodense vascular structures (red arrows). **B, D:** Corresponding CT-guided MWA demonstrates precise antenna placement (white arrows) with careful avoidance of critical vascular structures.

**Table 1:** Baseline patient and lesion characteristics.

**Characteristic**	**Total**
**Number of case (n=45)**	
Gender	
Male	35(77.8)
Female	10(22.2)
Age	59.9 ± 10.2
Positive for HBsAg	
Yes	39(86.7)
No	6(13.3)
BCLC stage	
A	35(77.8)
B	10(22.2)
Child-Pugh score	
A	38(84.4)
B	7(15.6)
ALBI degree	
1	25(55.6)
2	20(42.4)
AFP (ng/mL)	
≤200	37(82.2)
>200	8(17.8)
Number of tumors	
1	33(73.3)
2	12(26.7)
**Number of tumors (n=57)**	
Tumor diameter(mm)	18.2 ± 8.8
Tumor location	
II	3(5.3)
III	5(8.8)
IV	7(12.3)
V	11(19.3)
VI	7(12.3)
VII	12(21.0)
VIII	12(21.0)
Located in high-risk locations	
Yes	13(22.8)
No	44(77.2)

## Data Availability

The data and supportive information are available within the article.

## LIST OF ABBREVIATIONS

BCLC: Barcelona Clinic Liver Cancer
CBCT: Cone-Beam Computed Tomography
CEUS: Contrast-Enhanced Ultrasound
CT: Computed Tomography
DSA: Digital Subtraction Angiography
FOV: Field of View
HCC: Hepatocellular Carcinoma
HU: Hounsfield Unit(s)
IDR: Intrahepatic Distant Recurrence
LTP: Local Tumor Progression
MRI: Magnetic Resonance Imaging
MWA: Microwave Ablation
NCCT: Non-contrast Computed Tomography
OS: Overall Survival
RFS: Recurrence-Free Survival
SIR: Society of Interventional Radiology
TACE: Transarterial Chemoembolization
US: Ultrasound
WL: Window Level

## STANDARDS OF REPORTING

STROBE guidelines were followed.

## References

[1] Wang X., Gu D., Qin H., Lu X., Hu J., Zhang H., Wan X., He G. (2024). Prediction model and nomogram of early recurrence of hepatocellular carcinoma after ultrasound-guided microwave ablation.. Curr. Med. Imaging.

[2] Reig M., Sanduzzi-Zamparelli M., Forner A., Rimola J., Ferrer-Fabrega J., Burrel M., Garcia-Criado A., Diaz A., Llarch N., Iserte G., Molla M., Kelley R.K., Galle P.R. (2025). BCLC strategy for prognosis prediction and treatment recommendations: The 2026 update.. J. Hepatol..

[3] Singal A.G., Llovet J.M., Yarchoan M., Mehta N., Heimbach J.K., Dawson L.A., Jou J.H., Kulik L.M., Agopian V.G., Marrero J.A., Mendiratta-Lala M., Brown D.B., Rilling W.S., Goyal L., Wei A.C., Taddei T.H. (2023). AASLD practice guidance on prevention, diagnosis, and treatment of hepatocellular carcinoma.. Hepatology.

[4] Xie D., Shi J., Zhou J., Fan J., Gao Q. (2023). Clinical practice guidelines and real-life practice in hepatocellular carcinoma: A Chinese perspective.. Clin. Mol. Hepatol..

[5] Li B., Ren Z.W., Zhang C., Yu X.X., Xu X.X., Du Y., Yang H.F. (2024). Computed tomography-guided percutaneous cryoablation and microwave ablation in the treatment of perivascular hepatocellular carcinoma: A comparative study with propensity score matching.. Clin. Res. Hepatol. Gastroenterol..

[6] Crocetti L., de Baére T., Pereira P.L., Tarantino F.P. (2020). CIRSE standards of practice on thermal ablation of liver tumours.. Cardiovasc. Intervent. Radiol..

[7] Lin Z.Y., Fang Y., Chen J., Lin Q.F., Yan Y., Chen J., Li Y.L. (2020). Feasibility and efficacy study of microwave ablation of recurrent small HCC guided by enhanced liver-specific magnetic resonance imaging contrast agent.. Int. J. Hyperthermia.

[8] Li H., Vogl T.J., Chen K.A., Adwan H. (2025). A comparison of the efficacy and safety of US-, CT-, and MR-guided radiofrequency and microwave ablation for HCC: A systematic review and network meta-analysis.. Cancers.

[9] Zhang C., Qin Y., Song Y., Liu Y., Zhu X. (2024). Transarterial chemoembolization combined with microwave ablation in elderly patients with recurrent medium or large hepatocellular carcinoma.. J. Hepatocell. Carcinoma.

[10] Ji J., Yan L., Ma Y., Xu C., Zhou W.Z., Lv P. (2024). Transarterial chemoembolization combined with microwave ablation *versus* repeated resection for recurrent small hepatocellular carcinoma after resection: What is the better treatment?. Clin. Radiol..

[11] Wu C.H., Liang P.C., Su T.H., Lin M.C., Chang Y.H., Shih T.T.F., Kao J.H. (2021). Iodized oil computed tomography *versus* ultrasound-guided radiofrequency ablation for early hepatocellular carcinoma.. Hepatol. Int..

[12] Tan J., Mathy R.M., Chang D.H., Tang T., Zhang Z.S., Xiao Y.D. (2022). Combined transarterial iodized oil injection and computed tomography-guided thermal ablation for hepatocellular carcinoma: Utility of the iodized oil retention pattern.. Abdom. Radiol..

[13] Valls C., Figueras J., Bhattacjarya S., Dhillon A.P., Davidson B.R. (1998). Detection of hepatocellular carcinoma in cirrhotic livers with lipiodol-ct.. Hepatology.

[14] Paul S.B., Gamanagatti S.R., Dhamija E., Pulappadi V.P., Shalimar, Acharya S.K. (2022). Adapting the utility of lipiodol CT in treating small hepatocellular carcinoma.. J. Clin. Exp. Hepatol..

[15] May B.J., Charalel R.A. (2024). Cone beam computed tomography for the interventional oncologist: A practical approach.. Semin. Intervent. Radiol..

[16] Kim D.J., Chul-Nam I., Park S.E., Kim D.R., Lee J.S., Kim B.S., Choi G.M., Kim J., Won J.H. (2023). Added value of cone-beam computed tomography for detecting hepatocellular carcinomas and feeding arteries during transcatheter arterial chemoembolization focusing on radiation exposure.. Medicina.

[17] Abdelsalam M.E., Figueira T.M.A., Ensor J., Tam A.L., Avritscher R., Kaseb A., Gupta S. (2022). The impact of the use of C-arm cone-beam CT during chemoembolization for hepatocellular carcinoma.. Curr. Med. Imaging.

[18] Yan H., Xiang Z., Zhao C., Zou S., Huang M. (2023). Long-term outcomes of patients with hepatocellular carcinoma who underwent microwave ablation after downstaging with transarterial chemoembolization to barcelona clinic liver cancer stage A.. J. Vasc. Interv. Radiol..

[19] Hou Y.W., Zhang T.Q., Ma L.D., Jiang Y.Q., Han X., Di T., Tang L., Guo R.P., Chen M.S., Zhang J.X., Huang Z.M., Huang J.H. (2025). Long-term outcomes of transarterial chemoembolization plus ablation *versus* surgical resection in patients with large BCLC stage A/B HCC.. Acad. Radiol..

[20] Zhang Y.J., Chen M.S., Chen Y., Lau W.Y., Peng Z. (2021). Long-term outcomes of transcatheter arterial chemoembolization combined with radiofrequency ablation as an initial treatment for early-stage hepatocellular carcinoma.. JAMA Netw. Open.

